# Next Generation Exome Sequencing of Pediatric Asthma Identifies Rare and Novel Variants in Candidate Genes

**DOI:** 10.1155/2021/8884229

**Published:** 2021-02-08

**Authors:** Neda M. Bogari, Amr A. Amin, Husni H. Rayes, Ahmed Abdelmotelb, Mohiuddin M. Taher, Faisal A. Al-Allaf, Abdellatif Bouazzaoui, Luke O'Gorman, John W. Holloway

**Affiliations:** ^1^Faculty of Medicine, Department of Medical Genetics, Umm Al Qura University, Makkah, Saudi Arabia; ^2^Faculty of Medicine, Biochemistry Department, Umm Al Qura University, Makkah, Saudi Arabia; ^3^Faculty of Medicine, Ain Shams University, Cairo, Egypt; ^4^Maternity Children Hospital, Makkah, Saudi Arabia; ^5^Department of Pharmacology, Faculty of Medicine, University of Tanta, Tanta, Egypt; ^6^Science and Technology Unit, Umm Al-Qura University, Makkah, Saudi Arabia; ^7^Human Development and Health, Faculty of Medicine, University of Southampton, Southampton, UK

## Abstract

Multiple genes have been implicated to have a role in asthma predisposition by association studies. Pediatric patients often manifest a more extensive form of this disease and a particularly severe disease course. It is likely that genetic predisposition could play a more substantial role in this group. This study is aimed at identifying the spectrum of rare and novel variation in known pediatric asthma susceptibility genes using whole exome sequencing analysis in nine individual cases of childhood onset allergic asthma. DNA samples from the nine children with a history of bronchial asthma diagnosis underwent whole exome sequencing on Ion Proton. For each patient, the entire complement of rare variation within strongly associated candidate genes was catalogued. The analysis showed 21 variants in the subjects, 13 had been previously identified, and 8 were novel. Also, among of which, nineteen were nonsynonymous and 2 were nonsense. With regard to the novel variants, the 2 nonsynonymous variants in the *PRKG1* gene (*PRKG1*: p.C519W and *PRKG1*: p.G520W) were presented in 4 cases, and a nonsynonymous variant in the *MAVS* gene (*MAVS*: p.A45V) was identified in 3 cases. The variants we found in this study will enrich the variant spectrum and build up the database in the Saudi population. Novel eight variants were identified in the study which provides more evidence in the genetic susceptibility in asthma among Saudi children, providing a genetic screening map for the molecular genetic determinants of allergic disease in Saudi children, with the goal of reducing the impact of chronic diseases on the health and the economy. We believe that the advanced specified statistical filtration/annotation programs used in this study succeeded to release such results in a preliminary study, exploring the genetic map of that disease in Saudi children.

## 1. Introduction

Asthma and other allergic diseases, including allergic rhinitis, eczema, and food allergy, cause a substantial burden of disease in childhood. Although a rapid increase in asthma and allergies has been identified over the latter part of the 20th century, the reasons for this are still unknown [1]. Recent changes in environmental factors and their interactions with genetic profiles have been suggested as major factors responsible for the increase in asthma and allergic diseases [2].

Asthma, a chronic inflammatory respiratory condition characterized by hyperresponsive airways and reversible airflow obstruction, is a substantial public health problem that affects nearly 155 million individuals worldwide with the prevalence of current asthma and is higher in children compared than adults [3–5]. Although environmental factors are important, there are strong genetic predispositions for the development of allergic diseases. It has been reported that there are more than 100 candidate genes in every chromosome which are identified to have a linkage with asthma and the strength of association of these single-nucleotide polymorphisms (SNPs) with asthma varieties in different parts of the world [3–5]. A better knowledge of asthma susceptibility will hold promise for a better understanding of the pathology, diagnosis, prevention, treatment, and management of this increasingly frequent disease.

Next-generation sequencing (NGS) technology, in particular exome sequencing, currently represents the most powerful and cost effective approach to identifying variation in the human genome and has already been shown to uncover important disease-causing variation missed by GWAS studies [6–13]. Previous studies have implicated rare variants in asthma and asthma-related traits for a number of relevant genes [14, 15], suggesting that rare variants may indeed play an important role in asthma susceptibility.

Chromosome 17q21 was the first asthma susceptibility locus discovered by genome-wide association studies (GWAS) [10, 14, 16–19]. However, none of the genes within the locus had previously been implicated in asthma pathogenesis. SNPs in17q21 showing highly significant associations with childhood asthma correlated with the expression of ORMDL3 transcripts, suggesting ORMDL3 was a plausible asthma candidate gene in the locus [1]. Later, allele-specific gene expression was also observed for other genes in the 17q21 locus [20].

At least twelve GWAS of asthma have been conducted and have yielded numerous associations, with the most significant (and in most cases replicated) associations occurring in or near the following genes: ORMDL3 [1, 2], PDE4D [21], HLADRB1 [2], HLA-DQ [2, 3], RAD50-IL13 [3], DENND1B [4], TLE4 [5], SMAD3 [2], IL1RL1 [22], IL18R1 [2], IL33 [2], IL2RB [2], RORA [2], and SLC22A5 [2]. These findings have greatly expanded our understanding of the disease, having identified several novel genetic loci that had never previously been implicated in the pathogenesis of asthma (e.g., ORMDL3, RAD50, DENND1B, and TLE4). Despite these successes, no definitive causal variants have been identified in any of these genes. It is asserted that the associated variants are in LD with the causal variants in these genes, but more effort must be made to identify causal variants so that the biology of these genes in the etiology of asthma can be better understood.

Despite the success of GWAS in identifying the associated genetic variants for complex diseases and more than 1000 studies in the past few years conducted to identify the genetic complexity of many immune diseases including asthma, this approach still could—in part—explains the heritability of asthma regarding the clinical prediction of phenotypic heritability and immunological pathways [23].

This study is aimed at revealing the genetic determinants for pediatric asthma in Saudi Arabia using whole exome sequencing technique, reviewing the results with similar international studies.

## 2. Materials and Methods

### 2.1. Recruitment of Pediatric Asthma Cohort of Patients

Seventy-nine children included in this study were initially selected from the Pediatric Allergy and Immunology Clinic, Maternity Children Hospital Makkah, KSA, between Jan 2014 and Oct 2015. All children had been diagnosed with asthma after clinical evaluation in addition to both physiological assessment including pulmonary function tests (PFT) and the use of the International Study of Asthma and Allergies in Childhood (ISAAC) questionnaire (modified for the population) (note that PFT was not considered for young age who was not able to perform the test properly). They were aged between 5 and 14 years at the time of recruitment. Written informed consent was obtained from the attending parents of all the children. In the initial recruitment interview, clinical data and venous blood samples (3 ml of whole blood for CBC and DNA extraction and 3 ml for plasma separation) were collected.

Additional comprehensive clinical data were extracted from their medical records with their consent. For each patient, the information gathered included gender, dates of birth and initial diagnosis, laboratory investigations, physiological assessment, disease history, parents' history for any allergic and/or other autoimmune diseases, medication history (use of steroids, immunomodulators, and biological therapies), and history of potential allergen such as carpet, plants, and/or animal exposure.

### 2.2. Selection of Samples for Whole Exome Analysis

Nine asthmatic patients submitted for NGS were recruited based on both clinical evaluation and phenotypic criteria using the following:

- Global Initiative for Asthma (GINA) guidelines [24] in addition to the use of the International Study of Asthma and Allergies in Childhood (ISAAC) questionnaire (modified for the recruited patients) as initial identification with considerations of medications used (*β*-agonists and steroids)
- A history of respiratory symptoms such as shortness of breath, chest tightness, wheezing, and coughing that varies in intensity over time, as well as variable expiratory airflow limitation
- Physiological tests including both PFT and the spirometry measurements
- Only the patients not recorded with any other allergy symptoms—like skin or food allergy—who were submitted to whole exome sequencing (see (Table 1))

This study will be extended by performing genotyping for all selected patients for measuring the incidence of the identified variants.

### 2.3. DNA Extraction

Genomic DNA was extracted from EDTA anticoagulated peripheral venous blood samples using a spin column method using the recommended Applied Biosystem DNA extraction kit (catalogue number: K182001). All genomic DNA was quantified using a Nano-Drop 2000 spectrophotometer (Thermo Fisher Scientific). The DNA quality was assessed by the 260/280 ratio (1.8-2.0). For the library preparations, DNA was measured again using Qubit (Invitrogen) fluorometers, for adjusting all samples to 100 ng equally (accepted concentration was above 50 ng/*μ*l). Then, the libraries were purified using a magnetic plate and subsequently quantified using Ion Library TaqMan quantitation kit by real-time PCR.

### 2.4. Whole Exome Sequencing

Whole exome sequencing was performed for nine patients' DNA samples using Ion Torrent technology (Thermo Fisher Scientific). Libraries were prepared following the protocol described in Ion AmpliSeq Exome RDY library preparation user guide (Publication number MAN0010084) using Ion AmpliSeq Library Kit Plus (Cat. No. 4488990). One hundred nanograms of genomic DNA was used in library preparation with Ion AmpliSeq Exome RDY Kit 1×8 (Cat. No. A38262); then, the libraries were purified using a magnetic plate and subsequently quantified using Ion Library TaqMan quantitation kit (Part number 4468802) and Real-time PCR instrument 7500 Fast (Life Technologies, USA). Each sample was assigned a distinct barcode using an IonXpress barcode adapters 1–16 kit (Cat. No. 4471250). Barcoded libraries were diluted to 100 pM; three libraries were pooled and subjected to template preparation on Ion Sphere Particles (ISP) using the Ion OneTouch 2 System and the Ion PI Template OT2 Hi-Q Kit. Each templated ISP was loaded on the Ion PI v3 chip and sequenced on the Ion Proton instrument (Life Technologies, USA) using Ion PI Hi-Q sequencing Kit. Base calling and alignment were performed on a Torrent Suite v4.2 Server.

### 2.5. Informatic Analysis

Filtering low quality reads and removal of adapters were followed by alignment against the human reference genome (hg19 build). GATK v3.7 [25] was used to call SNPs and short indels in a multisample VCF file. GATK v3.7 variant quality score recalibration (VQSR) was applied to the dataset. Annotation was performed using AnnoVar [26] with a database of RefSeq transcripts [27], the Exome Aggregation Consortium (ExAC) [28] and pathogenicity scores including Sort Intolerant From Tolerant (SIFT) [29], PhyloP, Phast Cons [30], Genomic Evolutionary Rate Profiling (GERP++) [31], and Combined Annotation Dependent Depletion (CADD) [32].

Verify BamID v1.1.13 software was used to estimate possible contamination, and a “free mix” value threshold of 0.02 was applied [33]. Gene coverage was determined using SAM tools v1.3.1 [34] and BED tools v2.17.0 [35]. Variants with GATK quality score recalibration (VQSR) tranche >99% were excluded. Synonymous variants and variants which were found in ExAC (all populations) > 5% were also excluded from the analysis. Variants were prioritized by damaging pathogenicity scores (SIFT < 0.05, CADD phred > 15 [36], and GERP + +>2).

### 2.6. Selection of a Panel of Known Asthma Target Genes

All studies in the GWAS catalogue with “asthma” as a phenotype or keyword till 28 March 2017 were reviewed, and studies identifying asthma susceptibility variants that included pediatric subjects were identified and the significant loci retrieved. Following this, 16 additional genes from four studies were added following manual review of the literature. For a list of studies contributing to the candidate gene list (see Supplementary Table 3-3). In a total of twenty loci encompassing 131 potential candidate genes among which 110 genes were covered in this analysis.

## 3. Results

Verified BamIDf remix values for eight samples were below the 0.02 threshold and did not indicate substantial levels of contamination. One sample (subject 9) showed evidence for contamination and was excluded from further analysis. The minimum mean read depth was 47.7x, and the minimum coverage at 10x depth was 86.6% across the eight samples (Supplementary Table 2-3).

In a total of 8 subjects, 21 variants were identified following filtering for potential functionality, among which 13 had previously been identified in dbSNP and 8 were novel (Table 2). Nineteen of the 21 variants were non-synonymous and 2 were nonsense (stop gain). Among the 13 nonsynonymous variants in dbSNP, 2 variants have been previously studied. The rs3923647 was reported to be associated with increased production of the Th1 cytokines, IFN-*γ* and IL-2, following BCG vaccination [37]. The variant rs16889462 was identified and encoded for SLC30A8 gene reported for many functions in T2D pathophysiology involving the lowering of T2D risk in case of reduced SLC30A8 gene activity [38]. With regard to the novel variants, the 2 nonsynonymous variants in the PRKG1 gene (54041969 and 5404970) were present in 4 cases (1, 3, 4, and 5), and a nonsynonymous variant in the MAVS gene (3842992) was identified in 3 cases (4, 5, and 9).

## 4. Discussion

This report describes the results of a first study of exome sequencing in Saudi children diagnosed with asthma. Exome sequencing of eight children in this study has identified 21 potentially deleterious SNPs in known asthma genes; among them, eight novel variants and 13 variants previously deposited in dbSNP.

In this study, the genetic association was not repeated in the selected samples because this study was not designed as a family-based study. Our study was presented to define—for the first time in KSA—the genetic variants that are mostly associated with the asthma development in pediatric age in Makkah region through a large asthma GWAS cohort study, recognizing the possible genetic causes that implicated in asthma and perhaps also suggesting related biological pathways that play a role in the pathogenesis of asthma. Using advanced filtration and annotation programs, the total resulted variants were reduced to only 28 candidate genetic variants on 10 loci which were associated with childhood-onset disease.

On chromosome 1, three genetic variants were identified in three different samples (cases 3, 4, and 7) with two novel variants. One was reported previously, rs147827524, encoded for pyrin and HIN domain family member 1 gene (PYHIN1) that has been accounted for HIN200 proteins which are primarily nuclear proteins involved in transcriptional regulation of genes important for cell cycle control, differentiation, and apoptosis in addition to a surprisingly large proportion of asthma risk in people of African descent [39]. The two novel SNPs were discovered, rs152488110 and rs180651520, encoded for both cysteine-rich C-terminal 1 gene (CRCT1) and xenotropic and polytropic retrovirus receptor 1 gene (XPR1), respectively. The CRCT1 SNP is located near the end of the exon region in the gene and among many nonsynonymous reported SNPs. Hence, we supposed that it will be of nonsignificant effect on the protein function. However, the second variant, XPR1, has already been mentioned to play a role in modulating human airway smooth muscle (ASM) contraction, cell growth, and proinflammatory cytokine production that promote bronchoconstriction, airway inflammation, and remodeling in asthma [40]. On chromosome 2, only one variant was identified that was reported before, rs184451758, encoded for tensin 1 gene (TNS-1), the gene that has been accounted essentially for myofibroblast differentiation and extracellular matrix formation. The polymorphism in that gene is reported to be significantly associated with full form COPD risk [41]. On chromosome 4, three polymorphic variants were identified, one previously reported, rs3923647, encoded to Toll-like receptors1 (TLR1) polymorphisms that seems to play a role in susceptibility to asthma, atopic eczema, and allergic rhinitis [42]. The other two polymorphisms identified, rs745975778 and rs61529635, were reported for the same gene, synaptopodin 2 (SYNPO2), that was reported to be associated with total serum IgE in asthmatics in an independent GWAS, suggesting roles for this gene in asthma [43]. On chromosome 6, two closed sequentially novel variants were identified, rs32946010 and rs32946011, encoded for the same transcription factor element gene, bromodomain (BRD2), located on exon 8 (of 12 exons for that gene) where its protein has been shown to modulate transcription, in particular, in cell cycle-induced transcriptional activation. It has been reported recently that BRD2 protein inhibition attenuates neutrophil-dominant allergic airway disease in mice models [44]. On chromosome 8, one nonsynonymous polymorphic variant was identified, rs16889462, that was reported previously encoding one of the zinc efflux transporters, solute carrier family 30 member 8 (SLC30A8), which has been classified as one of the major components for providing zinc to insulin maturation and/or storage processes in insulin-secreting pancreatic *β*-cells. Five genome-wide association studies (GWAS) identified SLC30A8 polymorphism rs13266634 among Asian and European but not African populations [45]. On chromosome 9, only one reported polymorphism was identified, rs35642290, encoded for the cytoskeletal protein, talin 1 (TLN-1), which is considered one of the genes that might be associated with total IgE in asthmatics [46]. On chromosome 10, surprisingly, three variants in the same gene were identified including one previously reported SNP and two sequential novel ones, rs54041969 and rs54041970, identified in 50% of samples (cases 1, 3, 4, and 5) located on exon 14 (of 18 exons reported for that gene). All three variants encode the same protein kinase c GMP-dependent 1 gene (PRKG-1). All PRKG protein isoforms act as key mediators of the nitric oxide/cGMP signaling pathway and are important components of many signal transduction processes in diverse cell types [47]. It was reported that asthma is typically associated with high levels of exhaled nitric oxide (NO) which reduce the normal levels of S-nitrosothiols, which act as a bronchodilator in the airway [47].

On chromosome 12, one previously reported SNP, rs747186265, was identified—in only one sample (case number 6)—encoded for the otogelin-like gene/protein, which has been accounted to be expressed in the inner ear of vertebrates with the highest level of expression seen at the embryonic stage. No significant ear complications were recorded for that child in our study. We suppose that this variant was not significantly involved in the pathophysiology of asthma development. Also, we suppose that this SNP does not affect the protein structure and function even if it is classified as nonsynonymous, and/or we recommend performing further studies for those particular SNP effects. On chromosome 19, four polymorphisms were identified in our study including two previously reported SNPs, rs142299823 and rs74939505, encoded for both genes of zinc finger protein family, ZNF30 and ZNF154, respectively, involved in the process of DNA binding transcription factor activity. Additionally, two novel variants were identified both in two children in the analysis (cases 4 and 5), nonsynonymous (rs7267725) and stop gain (rs7267726). Both variants were encoded for the insulin receptor (INSR) gene region that was suggestively associated with asthma risk [48]. On chromosome 20, only one nonsynonymous previously reported variant, rs779234123, was identified—in three samples (cases 4, 5, and 8)—encoded for mitochondrial antiviral-signaling gene (MAVS), which expresses the protein required for protein kinase activity which is essential for gene expression. Impaired antiviral interferon expression may be involved in asthma exacerbations commonly caused by rhinovirus infections in asthmatic patients [49].

Numerous genetic and molecular studies have been carried out in the field of asthma in both the children and adults previously [50–60]. Among genetic studies, exome/NG sequencing studies have been documented throughout the global populations [61, 62]. The confirmed observations along with the genetic conclusions confirm the attractive genetic susceptibility factors in asthma patients. It is possible that genetic and nongenetic novel and documented variants might play a major role in the Saudi asthma patients. However, the limited patient's number and missing the screening of novel variants present a limitation of this study.

Not only genetic factors but also environmental, racial, and ethnic factors are associated with the asthma etiology; also, the dietary factors may have an active and direct impact on asthma symptoms [63–65]. Asthma can be triggered by exposure to many environmental factors [66]. Allergic asthma which is caused by allergens like pet dander, pests, dust mites, and mold is more common in children than nonallergic asthma. Nonallergic asthma can be caused by certain factors such as viruses, anxiety, stress, cold or dry air, and smoke. The incidence of allergic asthma is highest in childhood, while the incidence of nonallergic asthma is peaks in late adulthood [67]. It is reported that the African American and Hispanics are more susceptible to asthma, and the morbidity is high in this category [68, 69]. Children living in single-parent households are twice likely to be diagnosed with compared to children living of married parents [70]. However, to our knowledge, how these factors are contributing in the Arab population to asthma etiology is not fully understood, because limited studies are available regarding this disease from Middle Eastern countries.

### 4.1. Study Strengths and Limitations

The results of this study are unique genetic analyses of children with asthma in Saudi Arabia, identifying novel nonsynonymous polymorphisms that may correlate with severe asthma and weak response to treatment in children. This study will form the basis of future research in the field and would be of archival and perhaps diagnostic purposes.

This study has a limitation in terms of sample size also. Although we have initially recruited 79 cases with asthma in this study, due to budget constraints, we could do only 9 cases analysis by whole exome sequencing. This study has a limited number of patients and would benefit from larger sample size and perhaps comparison with genetic profiles in different countries.

## 5. Conclusions

In conclusion, this is the initial exome sequenced study implemented in the Saudi children diagnosed with asthma. Based on early studies and the results we found in this study, we assume that genetic variants might play a role in the increased susceptibility for the development of asthma. Other variants present in this study cannot be avoided considering the high number of loci and its specific genetic role involved within the disease in the global population. Future studies recommend to screen more patients for novel variants within the Saudi population to rule out its role in the asthma disease.

## Figures and Tables

**Table 1:** Summary of selected patients phenotypes and characteristics.

ID	Diagnosis	Gender	Age at diagnosis (year)	Blood eosinophil (%)	PFT	Treatment
					FEV1 % Predected	
1	BA	M	5.1	0.2	89%	*β*-Agonists and steroids
2	BA	F	5.9	0.22	90%	*β*-Agonists and steroids
3	BA/obese	M	8	0.26	89%	*β*-Agonists and steroids
4	BA	M	6	0.18	92%	*β*-Agonists and steroids
5	BA	M	5.5	0.41	83%	*β*-Agonists and steroids
6	BA	M	6.5	0.29	87%	*β*-Agonists and steroids
7	BA	M	6	7.57%	NA	*β*-Agonists and antihistaminic
8	BA (uncontrolled)	M	7.5	10.6%	NA	*β*-Agonists and steroids
9	BA	M	5	0.32	87%	*β*-Agonists and steroids

Table legend: phenotypic data of the patients (in addition to clinical and physiological assessment data including pulmonary function test, blood eosinophil percent, and the treatment used. PFT not performed for both samples 7 and 8). Note 1: sample 9 was selected but removed after Qc of genomic data. Note 2: the ID column includes the code number of the sample between brackets. ^∗^BA: bronchial asthma; ^∗^PFT: pulmonary function test.

**Table 2:** Twenty one shortlisted variants following the application of filter criteria for 8 Saudi Arabian pediatric asthma patients.

Chromosome	Location hg19	REF	ALT	Gene	Exonic. Func	Amino acid	dbSNP144	Allele frequency	Novel	1-sift	CADD phred	Gerp++	1	2	3	4	5	6	7	8
chr1	152488110	G	A	CRCT1	Nonsynonymous SNV	p.R84K		.	Yes	.	22.8	4.73			Het					
chr1	158908886	C	T	PYHIN1	Nonsynonymous SNV	p.S143F	rs147827524	0.0107		0.007	23.1	1.27				Het				
chr1	180651520	G	C	XPR1	Nonsynonymous SNV	p.A32P		.	Yes	0.013	30	5.71							Het	
chr2	218683301	C	T	TNS1	Nonsynonymous SNV	p.D1127N	rs184451758	0.0006		0.013	25.7	4.74						Het		
chr4	38799539	T	A	TLR1	Nonsynonymous SNV	p.H305L	rs3923647	0.0296		0.002	21.1	4.79		Het						
chr4	119952527	C	T	SYNPO2	Nonsynonymous SNV	p.P866L	rs745975778	2.47E-05		0.042	29.8	5.76	Het							
chr4	119978796	C	T	SYNPO2	Nonsynonymous SNV	p.R1134W	rs61529635	0.0057		0	26.2	3.99							Het	
chr6	32946010	G	T	BRD2	Nonsynonymous SNV	p.K562N		.	Yes	0.128	22.8	2.29							Het	
chr6	32946011	C	A	BRD2	Nonsynonymous SNV	p.H563N		.	Yes	0.304	20.5	5.24							Het	
chr6	165703388	A	T	C6orf118	Nonsynonymous SNV	p.I430N	rs202221904	4.97E-05		0.037	22.8	1.26							Het	
chr8	118184784	G	A	SLC30A8	Nonsynonymous SNV	p.R325Q	rs16889462	0.0163		0.099	19.95	-2.29								Het
chr9	35704426	C	T	TLN1	Nonsynonymous SNV	p.A1984T	rs35642290	0.0033		1	20.9	5	Het							
chr10	53822301	A	G	PRKG1	Nonsynonymous SNV	p.N267S	rs34997494	0.0445		0.053	23.3	5.76			Het					
chr10	54041969	T	G	PRKG1	Nonsynonymous SNV	p.C519W		.	Yes	0	29.1	5.49	Het		Het	Het	Het			
chr10	54041970	G	T	PRKG1	Nonsynonymous SNV	p.G520W		.	Yes	0	34	5.49	Het		Het	Het	Het			
chr12	80645886	G	A	OTOGL	Nonsynonymous SNV	p.R388Q	rs747186265	3.81E-05		0.064	35	5.95								
chr19	7267725	C	A	INSR	Nonsynonymous SNV	p.G95W		.	Yes	0	33	5.19				Het	Het			
chr19	7267726	A	C	INSR	Stop gain	p.Y94∗		.	Yes	.	32	-8.95				Het	Het			
chr19	35434582	G	T	ZNF30	Nonsynonymous SNV	p.G239W	rs142299823	0.0214		0.004	26.1	2.12		Het	Het					
chr19	58213743	G	A	ZNF154	Stop gain	p.R192∗	rs74939505	0.0088		.	24.9	-2.67				Het	Het			
chr20	3842992	C	T	MAVS	Nonsynonymous SNV	p.A45V	rs779234123	8.27E-06		0	26.9	3.22				Het	Het			Het

Chrom: chromosome; Location hg19: location of 5′ base of variant in hg19; REF: reference allele; ALT: alternative allele; Gene: gene symbol; Exonic. Func: consequence of the variant; Amino acid: amino acid change; dbSNP144: rsID in dbSNP144; Allele frequency: alternate allele frequency from ExAC database (all populations); Novel; 1-sift: sort intolerant from tolerant; CADD phred: Combined Annotation Dependent Depletion score (Phred scale); GERP++: Genomic Evolutionary Rate Profiling; sample IDs: “het” indicates a heterozygous genotype. Note: sample number 9 primarily selected was removed after Qc of genomic data.

## Data Availability

Data are available in the Supplementary Materials provided.

## Abbreviations

DNA: Deoxyribonucleic acid
EDTA: Ethylene Diamine tetra acetic acid
SNP: Single-nucleotide polymorphism
QC: Quality control
KSA: Kingdom of Saudi Arabia.

## Supplementary Materials

Supplementary 1 Table 1-3: mean depth, coverage at 10x depth across the target region and VerifyBamID freemix values for each sample.

Supplementary 2 Table 2-3: gene coverage for 107 genes from the asthma gene panel.

Supplementary 3 Table 3-3: list of selected genes associated with asthma.
