# Supplementary material for: Next Generation Exome Sequencing of Pediatric Asthma Identifies Rare and Novel Variants in Candidate Genes
Source: Dis Markers. 2021 Feb 8;2021:8884229. doi: 10.1155/2021/8884229 (PMC7888305; doi:10.1155/2021/8884229)
Supplement: Supplementary 1 — Table 1-3: mean depth, coverage at 10x depth across the target region and VerifyBamID freemix values for each sample. [file 8884229.f1.docx]

Additional File 1

| Table 1: Mean depth, coverage at 10x depth across target region and VerifyBamID freemix values for each sample. **Sample ID** | **Mean depth** | **Coverage of target region at 10x (%)** | **VerifyBamID Freemix** |
| --- | --- | --- | --- |
| 1 | 47.7 | 86.6 | 0.0014 |
| 2 | 118.3 | 93.1 | 0.0003 |
| 3 | 89.6 | 91.2 | 0.0001 |
| 4 | 101.6 | 93.2 | 0.0004 |
| 5 | 178.3 | 94.7 | 0.0000 |
| 6 | 88.9 | 91.5 | 0.0019 |
| 7 | 53.9 | 87.7 | 0.0016 |
| 8 | 59.3 | 87.8 | 0.0002 |

Additional File 2

Table 2: Gene coverage for 107 genes from the asthma gene panel.

| **Gene** | **1x** | **5x** | **10x** | **20x** | **30x** | **50x** |
| --- | --- | --- | --- | --- | --- | --- |
| ***ABI3BP*** | 0.996 | 0.990 | 0.930 | 0.773 | 0.662 | 0.417 |
| ***ACO1*** | 0.996 | 0.996 | 0.993 | 0.953 | 0.944 | 0.812 |
| ***ADAMTS9*** | 0.997 | 0.957 | 0.953 | 0.899 | 0.821 | 0.647 |
| ***AGFG1*** | 0.997 | 0.884 | 0.830 | 0.772 | 0.670 | 0.319 |
| ***ARPP21*** | 0.996 | 0.995 | 0.989 | 0.881 | 0.840 | 0.685 |
| ***ATG3*** | 0.996 | 0.932 | 0.812 | 0.673 | 0.435 | 0.279 |
| ***BRD2*** | 0.998 | 0.987 | 0.958 | 0.886 | 0.840 | 0.774 |
| ***C5orf51*** | 0.996 | 0.989 | 0.947 | 0.775 | 0.533 | 0.293 |
| ***C6orf118*** | 0.997 | 0.996 | 0.993 | 0.950 | 0.781 | 0.543 |
| ***CDH13*** | 0.996 | 0.996 | 0.990 | 0.927 | 0.825 | 0.659 |
| ***CDHR3*** | 0.996 | 0.989 | 0.971 | 0.951 | 0.889 | 0.751 |
| ***CDK2*** | 0.996 | 0.996 | 0.989 | 0.944 | 0.835 | 0.688 |
| ***CLEC16A*** | 0.996 | 0.959 | 0.938 | 0.881 | 0.799 | 0.685 |
| ***CNTN5*** | 0.996 | 0.994 | 0.934 | 0.849 | 0.720 | 0.603 |
| ***COL22A1*** | 0.996 | 0.980 | 0.955 | 0.914 | 0.841 | 0.682 |
| ***CRB1*** | 0.998 | 0.989 | 0.983 | 0.925 | 0.875 | 0.728 |
| ***CRCT1*** | 0.998 | 0.611 | 0.282 | 0.137 | 0.073 | 0.000 |
| ***CRIM1*** | 0.997 | 0.994 | 0.977 | 0.973 | 0.947 | 0.889 |
| ***DCLK1*** | 0.996 | 0.996 | 0.995 | 0.984 | 0.925 | 0.713 |
| ***ERBB4*** | 0.996 | 0.996 | 0.990 | 0.885 | 0.772 | 0.518 |
| ***FAM19A2*** | 0.995 | 0.995 | 0.993 | 0.939 | 0.758 | 0.747 |
| ***GAB1*** | 0.998 | 0.937 | 0.863 | 0.703 | 0.602 | 0.519 |
| ***GAS1*** | 0.999 | 0.260 | 0.224 | 0.161 | 0.012 | 0.000 |
| ***GAS8*** | 0.997 | 0.964 | 0.910 | 0.838 | 0.771 | 0.539 |
| ***GC*** | 0.996 | 0.996 | 0.994 | 0.972 | 0.897 | 0.807 |
| ***GSDMA*** | 0.996 | 0.995 | 0.989 | 0.938 | 0.835 | 0.686 |
| ***GSDMB*** | 0.997 | 0.996 | 0.996 | 0.995 | 0.987 | 0.919 |
| ***HLA-DOA*** | 0.997 | 0.992 | 0.967 | 0.832 | 0.798 | 0.634 |
| ***HLA-DPA1*** | 0.997 | 0.997 | 0.997 | 0.997 | 0.994 | 0.981 |
| ***HLA-DQA2*** | 0.997 | 0.718 | 0.571 | 0.542 | 0.539 | 0.519 |
| ***HLA-DQB1*** | 0.995 | 0.707 | 0.516 | 0.466 | 0.378 | 0.244 |
| ***HLA-DRA*** | 0.996 | 0.995 | 0.986 | 0.915 | 0.873 | 0.593 |
| ***HLA-DRB1*** | 0.995 | 0.286 | 0.223 | 0.065 | 0.000 | 0.000 |
| ***HPSE2*** | 0.996 | 0.996 | 0.994 | 0.989 | 0.957 | 0.814 |
| ***IGSF3*** | 0.998 | 0.397 | 0.338 | 0.294 | 0.287 | 0.229 |
| ***IKZF1*** | 0.997 | 0.997 | 0.996 | 0.933 | 0.834 | 0.593 |
| ***IKZF3*** | 0.997 | 0.911 | 0.910 | 0.891 | 0.795 | 0.545 |
| ***IKZF4*** | 0.997 | 0.996 | 0.975 | 0.892 | 0.774 | 0.538 |
| ***IL13*** | 0.996 | 0.995 | 0.995 | 0.993 | 0.969 | 0.816 |
| ***IL18R1*** | 0.997 | 0.997 | 0.994 | 0.938 | 0.707 | 0.472 |
| ***IL1RL1*** | 0.997 | 0.983 | 0.947 | 0.916 | 0.833 | 0.672 |
| ***IL2RB*** | 0.997 | 0.959 | 0.953 | 0.914 | 0.824 | 0.669 |
| ***IL33*** | 0.995 | 0.898 | 0.848 | 0.844 | 0.832 | 0.692 |
| ***IL5*** | 0.996 | 0.996 | 0.996 | 0.986 | 0.811 | 0.157 |
| ***IL6R*** | 0.996 | 0.996 | 0.990 | 0.884 | 0.669 | 0.577 |
| ***INSR*** | 0.997 | 0.966 | 0.959 | 0.836 | 0.797 | 0.702 |
| ***IRF1*** | 0.997 | 0.996 | 0.995 | 0.990 | 0.820 | 0.739 |
| ***JRKL*** | 0.999 | 0.999 | 0.998 | 0.995 | 0.974 | 0.725 |
| ***KLHL5*** | 0.997 | 0.994 | 0.945 | 0.846 | 0.720 | 0.428 |
| ***KRT25*** | 0.997 | 0.918 | 0.911 | 0.814 | 0.690 | 0.465 |
| ***LCE3E*** | 0.998 | 0.719 | 0.597 | 0.595 | 0.511 | 0.000 |
| ***LMO4*** | 0.996 | 0.992 | 0.913 | 0.783 | 0.780 | 0.731 |
| ***LRRC32*** | 0.999 | 0.943 | 0.942 | 0.908 | 0.839 | 0.777 |
| ***LRRC3C*** | 0.998 | 0.786 | 0.760 | 0.639 | 0.613 | 0.472 |
| ***MAVS*** | 0.998 | 0.998 | 0.996 | 0.950 | 0.936 | 0.760 |
| ***MKLN1*** | 0.996 | 0.981 | 0.909 | 0.744 | 0.616 | 0.346 |
| ***MTHFR*** | 0.997 | 0.995 | 0.965 | 0.930 | 0.863 | 0.772 |
| ***NOTCH4*** | 0.998 | 0.994 | 0.973 | 0.913 | 0.836 | 0.671 |
| ***OTOGL*** | 0.996 | 0.964 | 0.916 | 0.800 | 0.654 | 0.406 |
| ***OXCT1*** | 0.996 | 0.991 | 0.953 | 0.806 | 0.744 | 0.566 |
| ***PBX2*** | 0.997 | 0.841 | 0.782 | 0.750 | 0.743 | 0.637 |
| ***PDE4D*** | 0.997 | 0.899 | 0.887 | 0.821 | 0.709 | 0.515 |
| ***PRKG1*** | 0.996 | 0.938 | 0.886 | 0.852 | 0.748 | 0.529 |
| ***PSAP*** | 0.996 | 0.996 | 0.989 | 0.939 | 0.923 | 0.776 |
| ***PTCHD3*** | 0.999 | 0.942 | 0.928 | 0.851 | 0.759 | 0.593 |
| ***PTHLH*** | 0.998 | 0.994 | 0.978 | 0.836 | 0.785 | 0.654 |
| ***PYHIN1*** | 0.997 | 0.995 | 0.977 | 0.902 | 0.746 | 0.534 |
| ***RAB18*** | 0.997 | 0.975 | 0.917 | 0.835 | 0.644 | 0.437 |
| ***RAD50*** | 0.997 | 0.973 | 0.943 | 0.841 | 0.613 | 0.310 |
| ***RANBP6*** | 1.000 | 0.701 | 0.665 | 0.622 | 0.581 | 0.483 |
| ***RAP1GAP2*** | 0.996 | 0.992 | 0.965 | 0.879 | 0.761 | 0.543 |
| ***RBM17*** | 0.996 | 0.994 | 0.972 | 0.817 | 0.775 | 0.635 |
| ***RORA*** | 0.996 | 0.940 | 0.754 | 0.744 | 0.734 | 0.542 |
| ***RREB1*** | 0.998 | 0.934 | 0.885 | 0.792 | 0.640 | 0.401 |
| ***SCG3*** | 0.996 | 0.995 | 0.989 | 0.933 | 0.858 | 0.605 |
| ***SCML4*** | 0.997 | 0.997 | 0.996 | 0.994 | 0.976 | 0.795 |
| ***SEMA3E*** | 0.997 | 0.995 | 0.988 | 0.910 | 0.756 | 0.555 |
| ***SGMS1*** | 0.997 | 0.997 | 0.997 | 0.986 | 0.942 | 0.787 |
| ***SLC22A5*** | 0.997 | 0.959 | 0.875 | 0.724 | 0.657 | 0.410 |
| ***SLC25A46*** | 0.997 | 0.955 | 0.856 | 0.730 | 0.589 | 0.273 |
| ***SLC30A8*** | 0.996 | 0.996 | 0.995 | 0.985 | 0.954 | 0.731 |
| ***SLC8A1*** | 0.998 | 0.998 | 0.995 | 0.973 | 0.880 | 0.720 |
| ***SMAD3*** | 0.997 | 0.832 | 0.772 | 0.761 | 0.688 | 0.427 |
| ***SPATS2L*** | 0.997 | 0.997 | 0.996 | 0.982 | 0.881 | 0.633 |
| ***SYNPO2*** | 0.999 | 0.998 | 0.991 | 0.953 | 0.900 | 0.743 |
| ***TBC1D4*** | 0.997 | 0.981 | 0.945 | 0.816 | 0.743 | 0.623 |
| ***THUMPD2*** | 0.996 | 0.991 | 0.811 | 0.674 | 0.550 | 0.422 |
| ***TLN1*** | 0.999 | 0.972 | 0.953 | 0.918 | 0.904 | 0.857 |
| ***TLR1*** | 1.000 | 0.802 | 0.802 | 0.799 | 0.774 | 0.642 |
| ***TNS1*** | 0.997 | 0.989 | 0.978 | 0.902 | 0.847 | 0.719 |
| ***TSLP*** | 0.997 | 0.997 | 0.997 | 0.993 | 0.975 | 0.842 |
| ***TYRP1*** | 0.997 | 0.997 | 0.990 | 0.855 | 0.815 | 0.602 |
| ***VAV3*** | 0.996 | 0.971 | 0.966 | 0.884 | 0.700 | 0.468 |
| ***WDR36*** | 0.997 | 0.977 | 0.942 | 0.832 | 0.688 | 0.470 |
| ***XKR6*** | 0.998 | 0.855 | 0.781 | 0.647 | 0.529 | 0.473 |
| ***XPR1*** | 0.997 | 0.959 | 0.909 | 0.869 | 0.817 | 0.593 |
| ***ZFYVE28*** | 0.997 | 0.903 | 0.873 | 0.729 | 0.573 | 0.325 |
| ***ZNF154*** | 0.998 | 0.959 | 0.949 | 0.904 | 0.882 | 0.749 |
| ***ZNF30*** | 0.998 | 0.998 | 0.998 | 0.982 | 0.804 | 0.520 |
| ***ZNF665*** | 0.999 | 0.998 | 0.980 | 0.906 | 0.794 | 0.566 |
| ***ZNF71*** | 1.000 | 0.924 | 0.833 | 0.699 | 0.471 | 0.409 |
| ***ZNF766*** | 0.998 | 0.996 | 0.980 | 0.879 | 0.779 | 0.753 |

Additional File 3

Table 3 : list of selected genes associated with asthma

| **Number** | **Gene** | **FIRST AUTHOR** | **REGION** | **SNPS** | **P-VALUE** |
| --- | --- | --- | --- | --- | --- |
|  | GAB1 | Hirota T [64] | 4q31.21 | rs3805236 | 7.00E-08 |
|  | TSLP | Hirota T [64] | 5q22.1 | rs1837253 | 1.00E-16 |
|  |  | Torgerson DG [65] | 5q22.1 | rs1837253 | 1.00E-14 |
|  |  | Ferreira MA [66] | 5q22.1 | rs1837253 | 1.00E-09 |
|  | PBX2 | Hirota T [64] | 6p21.32 | rs204993 | 2.00E-15 |
|  | NOTCH4 | Hirota T [64] | 6p21.32 | rs404860 | 4.00E-23 |
|  | BRD2 | Hirota T [64] | 6p21.32 | rs9500927 | 4.00E-09 |
|  |  | [Leusink M](https://www.ncbi.nlm.nih.gov/pubmed/?term=Leusink%20M%5BAuthor%5D&cauthor=true&cauthor_uid=25963336) [67] | 6p21.32 | rs10484568 |  |
|  |  | [Leusink M](https://www.ncbi.nlm.nih.gov/pubmed/?term=Leusink%20M%5BAuthor%5D&cauthor=true&cauthor_uid=25963336) [67] | 6p21.32 | rs10484568 |  |
|  | CDK2 | Hirota T [64] | 12q13.2 | rs2069408 | 1.00E-10 |
|  | IKZF4 | Hirota T [64] | 12q13.2 | rs1701704 | 2.00E-13 |
|  | SLC30A8 | Noguchi E [68] | 8q24.11 | rs3019885 | 5.00E-13 |
|  | IL18R1 | Moffatt MF [11] | 2q12.1 | rs3771166 | 3.00E-09 |
|  |  | Wan YI [69] | 2q12.1 | rs9807989 | 6.00E-08 |
|  |  | Barreto-Luis A [70] | 2q12.1 | rs10197862 | 2.00E-06 |
|  |  | Ramasamy A [71] | 2q12.1 | rs13408661 | 1.00E-09 |
|  | PRKG1 | Ferreira MA [68] | 10q21.1 | rs7922491 | 5.00E-07 |
|  | IL6R | Ferreira MA [68] | 1q21.3 | rs4129267 | 2.00E-08 |
|  | SMAD3 | Moffatt MF [11] | 15q22.33 | rs744910 | 4.00E-09 |
|  |  | Ferreira MA [66] | 15q22.33 | rs17294280 | 4.00E-09 |
|  | GSDMA | Moffatt MF [11] | 17q21.1 | rs3894194 | 5.00E-09 |
|  |  | Ferreira MA [66] | 17q21.1 | rs7212938 | 4.00E-10 |
|  |  | Bonnelykke K [72] | 17q21.1 | rs3894194 | 3.00E-21 |
|  | IL2RB | Moffatt MF [11] | 22q12.3 | rs2284033 | 1.00E-08 |
|  | SLC22A5 | Moffatt MF [11] | 5q31.1 | rs2073643 | 2.00E-07 |
|  | IL13 | Moffatt MF [11] | 5q31.1 | rs1295686 | 1.00E-07 |
|  |  | Bonnelykke K [72] | 5q31.1 | rs1295686 | 2.00E-06 |
|  | RORA | Moffatt MF [11] | 15q22.2 | rs11071559 | 1.00E-07 |
|  | LRRC3C | Ferreira MA [73] | 17q21.1 | rs6503525 | 5.00E-07 |
|  |  | Wan YI [69] | 17q21.1 | rs4794820 | 1.00E-08 |
|  | ACO1 | Wan YI [69] | 9p21.1 | rs10970976 | 4.00E-06 |
|  | ZNF665 | Wan YI [69] | 19q13.42 | rs16984547 | 4.00E-06 |
|  | CRB1 | Sleiman PM [74] | 1q31.3 | rs2786098 | 2.00E-13 |
|  |  | Sleiman PM [74] | 1q31.3 | rs2786098 | 9.00E-11 |
|  | RAD50 | Li X [75] | 5q31.1 | rs2244012 | 3.00E-07 |
|  |  | Bonnelykke K [72] | 5q31.1 | rs6871536 | 8.00E-07 |
|  | SCG3 | Li X [75] | 15q21.2 | rs17525472 | 2.00E-06 |
|  | SPATS2L | Himes BE [76] | 2q33.1 | rs295137 | 1.00E-06 |
|  | ADAMTS9 | Barreto-Luis A [70] | 3p14.1 | rs9866261 | 1.00E-07 |
|  | CRIM1 | Kim JH [77] | 2p22.2 | rs848512 | 1.00E-06 |
|  | ZNF71 | Kim JH [77] | 19q13.43 | rs10404342 | 8.00E-06 |
|  | TLN1 | Kim JH [77] | 9p13.3 | rs4879926 | 8.00E-06 |
|  | SYNPO2 | Kim JH [77] | 4q26 | rs1472066 | 8.00E-06 |
|  | LCE3E | Torgerson DG [65] | 1q21.3 | rs4845783 | 6.00E-06 |
|  | CDH13 | Kim JH [77] | 16q23.3 | rs6563898 | 8.00E-06 |
|  | PDE4D | Himes BE [78] | 5q12.1 | rs1588265 | 3.00E-08 |
|  | PYHIN1 | Torgerson DG [65] | 1q23.1 | rs1101999 | 4.00E-09 |
|  | RANBP6 | Torgerson DG [65] | 9p24.1 | rs2381416 | 2.00E-12 |
|  |  | Moffatt MF [11] | 9p24.1 | rs1342326 | 9.00E-10 |
|  |  | Ferreira MA [66] | 9p24.1 | rs72699186 | 2.00E-09 |
|  |  | Ferreira MA [66] | 9p24.1 | rs343496 | 2.00E-06 |
|  | CRCT1 | Torgerson DG [65] | 1q21.3 | rs4845783 | 6.00E-06 |
|  | HLA-DPA1 | Noguchi E [79] | 6p21.32 | rs987870 | 2.00E-10 |
|  | IRF1 | Myers RA [80] | 5q31.1 | rs2549003 | 9.00E-07 |
|  | MAVS | Li X [75] | 20p13 | rs4815617 | 8.00E-06 |
|  | ERBB4 | Myers RA [80] | 2q34 | rs4673659 | 9.00E-07 |
|  | C6orf118 | Myers RA [80] | 6q27 | rs2675724 | 2.00E-07 |
|  | RAP1GAP2 | Myers RA [80] | 17p13.3 | rs9895098 | 3.00E-07 |
|  | TLR1 | Ferreira MA [66] | 4p14 | rs4833095 | 5.00E-12 |
|  | WDR36 | Ferreira MA [66] | 5q22.1 | rs1438673 | 3.00E-11 |
|  | CLEC16A | Ferreira MA [66] | 16p13.13 | rs62026376 | 1.00E-08 |
|  | SLC25A46 | Ferreira MA [66] | 5q22.1 | rs3853750 | 2.00E-07 |
|  | PTHLH | Ferreira MA [66] | 12p11.22 | rs11049300 | 3.00E-07 |
|  | IKZF3 | Ferreira MA[66] | 17q21.1 | rs12450323 | 4.00E-07 |
|  | XKR6 | Ferreira MA [66] | 8p23.1 | rs6982751 | 4.00E-07 |
|  | RBM17 | Ferreira MA [66] | 10p15.1 | rs41295115 | 5.00E-07 |
|  | TNS1 | Ferreira MA [66] | 2q35 | rs76043829 | 6.00E-07 |
|  | VAV3 | Ferreira MA [66] | 1p13.3 | rs7521681 | 7.00E-07 |
|  | GAS1 | Ding L [81] | 9q21.33 | rs11141597 | 2.00E-06 |
|  | FAM19A2 | Ferreira MA[66] | 12q14.1 | rs17605016 | 2.00E-06 |
|  | HLA-DRB1 | Lasky-Su J [82] | 6p21.32 | rs9272346 | 2.00E-08 |
|  | COL22A1 | Duan QL [83] | 8q24.23 | rs6988229 | 9.00E-06 |
|  | RAB18 | White MJ [84] | 10p12.1 | rs660498 | 2.00E-07 |
|  | DCLK1 | Forno E [85] | 13q13.3 | rs7328278 | 3.00E-06 |
|  | PTCHD3 | White MJ [84] | 10p12.1 | rs660498 | 2.00E-07 |
|  | SEMA3E | White MJ [84] | 7q21.11 | rs17446324 | 5.00E-06 |
|  | INSR | White MJ [84] | 19p13.2 | rs67731056 | 7.00E-06 |
|  | TYRP1 | Ding L [81] | 9p23 | rs16929097 | 8.00E-09 |
|  | CDHR3 | Bonnelykke K [72] | 7q22.3 | rs6967330 | 3.00E-14 |
|  | IGSF3 | Ding L [81] | 1p13.1 | rs17036023 | 5.00E-06 |
|  | HPSE2 | Ding L [81] | 10q24.2 | rs12570188 | 5.00E-08 |
|  | PSAP | Ding L [81] | 10q22.1 | rs11000019 | 8.00E-08 |
|  | ATG3 | Ding L [81] | 3q13.2 | rs2705520 | 2.00E-06 |
|  | ARPP21 | Ding L [81] | 3p22.3 | rs17033506 | 4.00E-07 |
|  | SLC8A1 | Ding L [81] | 2p22.1 | rs6721181 | 6.00E-07 |
|  | THUMPD2 | Ding L [81] | 2p22.1 | rs6721181 | 6.00E-07 |
|  | MKLN1 | Ding L [81] | 7q32.3 | rs7807274 | 4.00E-06 |
|  | XPR1 | Ding L [81] | 1q25.3 | rs7527074 | 9.00E-06 |
|  | IL33 | Torgerson DG [65] | 9p24.1 | rs2381416 | 2.00E-12 |
|  |  | Moffatt MF [11] | 9p24.1 | rs1342326 | 9.00E-10 |
|  |  | Ferreira MA [66] | 9p24.1 | rs72699186 | 2.00E-09 |
|  |  | Bonnelykke K [72] | 9p24.1 | rs928413 | 9.00E-13 |
|  | ABI3BP | Ding L [81] | 3q12.2 | rs9823506 | 6.00E-08 |
|  | KLHL5 | Ding L [81] | 4p14 | rs35141484 | 3.00E-07 |
|  | JRKL | [Leusink M](https://www.ncbi.nlm.nih.gov/pubmed/?term=Leusink%20M%5BAuthor%5D&cauthor=true&cauthor_uid=25963336) [67] |  | rs921561 | 2.56 × 10^−5^ |
|  | OXCT1 | [Leusink M](https://www.ncbi.nlm.nih.gov/pubmed/?term=Leusink%20M%5BAuthor%5D&cauthor=true&cauthor_uid=25963336) [67] |  | rs151191974 |  |
|  | AGFG1 | Himes BE | 2q36.3 | rs6731443 | 2.00E-06 |
|  | GC | Lasky-Su J [86] | 4q13.3 | rs2282679 | 2.00E-14 |
|  | *RREB1* | [Leusink M](https://www.ncbi.nlm.nih.gov/pubmed/?term=Leusink%20M%5BAuthor%5D&cauthor=true&cauthor_uid=25963336) [67] |  | rs35742417 | 2.44 × 10^−6^ |
|  | *GAS8* |  |  | rs117053233 | 2.63 × 10−5 6.14 × 10−5 |
|  | *KRT25* | [Leusink M](https://www.ncbi.nlm.nih.gov/pubmed/?term=Leusink%20M%5BAuthor%5D&cauthor=true&cauthor_uid=25963336) [67] |  | rs72821893 | 6.21 × 10−5 |
|  | *IKZF1* | [Leusink M](https://www.ncbi.nlm.nih.gov/pubmed/?term=Leusink%20M%5BAuthor%5D&cauthor=true&cauthor_uid=25963336) [67] |  | rs1456896 | 8.80 × 10−5 |
|  | *CNTN5* | [Leusink M](https://www.ncbi.nlm.nih.gov/pubmed/?term=Leusink%20M%5BAuthor%5D&cauthor=true&cauthor_uid=25963336) [67] |  | rs921561 | 2.56 × 10^−5^ |
|  | *OTOGL* | [Leusink M](https://www.ncbi.nlm.nih.gov/pubmed/?term=Leusink%20M%5BAuthor%5D&cauthor=true&cauthor_uid=25963336) [67] |  | rs1551120 | 2.67 × 10−5 |
|  | *TBC1D4* | [Leusink M](https://www.ncbi.nlm.nih.gov/pubmed/?term=Leusink%20M%5BAuthor%5D&cauthor=true&cauthor_uid=25963336) [67] |  | rs716655 | 5.56 × 10−5 |
|  | *SCML4* | [Leusink M](https://www.ncbi.nlm.nih.gov/pubmed/?term=Leusink%20M%5BAuthor%5D&cauthor=true&cauthor_uid=25963336) [67] |  | rs847005 | 4.49 × 10−5 |
|  | *ZNF30* | [Leusink M](https://www.ncbi.nlm.nih.gov/pubmed/?term=Leusink%20M%5BAuthor%5D&cauthor=true&cauthor_uid=25963336) [67] |  | rs142299823 | 4.60 × 10−5 |
|  | *SGMS1* | [Leusink M](https://www.ncbi.nlm.nih.gov/pubmed/?term=Leusink%20M%5BAuthor%5D&cauthor=true&cauthor_uid=25963336) [67] |  | rs2574951 | 4.68 × 10−5 |
|  | *LMO4* | [Leusink M](https://www.ncbi.nlm.nih.gov/pubmed/?term=Leusink%20M%5BAuthor%5D&cauthor=true&cauthor_uid=25963336) [67] |  | rs4655852 |  |
|  | *ZNF766* | [Leusink M](https://www.ncbi.nlm.nih.gov/pubmed/?term=Leusink%20M%5BAuthor%5D&cauthor=true&cauthor_uid=25963336) [67] |  | rs12462608 | 5.18 × 10−5 |
|  | *C5orf51* | [Leusink M](https://www.ncbi.nlm.nih.gov/pubmed/?term=Leusink%20M%5BAuthor%5D&cauthor=true&cauthor_uid=25963336) [67] |  | rs151191974 | 6.44 × 10−5 |
|  | *ZNF154* | [Leusink M](https://www.ncbi.nlm.nih.gov/pubmed/?term=Leusink%20M%5BAuthor%5D&cauthor=true&cauthor_uid=25963336) [67] |  | rs34282745 | 8.13 × 10−5 |
|  | *ZFYVE28* | [Leusink M](https://www.ncbi.nlm.nih.gov/pubmed/?term=Leusink%20M%5BAuthor%5D&cauthor=true&cauthor_uid=25963336) [67] |  | rs17768776 | 8.69 × 10−5 |
|  | MTHFR | [Igartua C](https://www.ncbi.nlm.nih.gov/pubmed/?term=Igartua%20C%5BAuthor%5D&cauthor=true&cauthor_uid=25591454) [87] |  | rs1801133 | 9.30 × 10−5 |
|  | IL5 | Myers RA [80] | 5q31.1 | rs2549003 | 9.00E-07 |
|  | LRRC32 | Ferreira MA [68] | 11q13.5 | rs7130588 | 2.00E-08 |
|  | GSDMB | Moffatt MF [11] | 17q21.1 | rs7216389 | 9.00E-11 |
|  |  | Moffatt MF [11] | 17q21.1 | rs2305480 | 1.00E-07 |
|  |  | Torgerson DG [65] | 17q21.1 | rs11078927 | 2.00E-16 |
|  |  | Bonnelykke K [72] | 17q21.1 | rs2305480 | 6.00E-23 |
|  |  |  | 17q12–21 | rs2305480 |  |
|  |  |  | 17q12–21 | rs7216389 |  |
|  | HLA-DOA (BRD2 ) | Hirota T [64] | 6p21.32 | rs9500927 | 4.00E-09 |
|  | HLA-DOA  (BRD2) | [Leusink M](https://www.ncbi.nlm.nih.gov/pubmed/?term=Leusink%20M%5BAuthor%5D&cauthor=true&cauthor_uid=25963336) [67] |  | rs10484568 | 7.42 × 10−5 |
|  | HLA-DQA2 | Hirota T [64] | 6p21.32 | rs9275698 | 5.00E-12 |
|  | HLA-DQA1 (HLA-DQB1) | Moffatt MF [22, 88]. | 6p21.32 | rs9273349 | 7.00E-14 |
|  |  | Ferreira MA [66] | 6p21.32 | rs9273373 | 4.00E-14 |
|  | HLA-DQB1 (MTCO3P1) | Hirota T [64] | 6p21.32 | rs7775228 | 5.00E-15 |
|  | BTNL2  (HLA-DRA) | Ramasamy A [71] | 6p21.32 | rs9268516 | 1.00E-08 |
|  | HLA-DRA (HLA-DRB9) | Hirota T [64] | 6p21.32 | rs3129890 | 5.00E-13 |
|  | IL1RL1 | Ramasamy A [71] | 2q12.1 | rs13408661 | 1.00E-09 |
|  |  | Barreto-Luis A [70] | 2q12.1 | rs10197862 | 2.00E-06 |
|  |  | Torgerson DG [65] | 2q12.1 | rs3771180 | 2.00E-15 |
|  |  | Ferreira MA [66] | 2q12.1 | rs10197862 | 4.00E-11 |
|  | IL1RL1 - IL18R1 | Wan YI [69] | 2q12.1 | rs9807989 | 6.00E-08 |
|  | C20orf29  (AP5S1)(MAVS ) | Li X [75] | 20p13 | rs4815617 | 8.00E-06 |
|  | BCL2  (BCLAF1P1  ) |  |  |  |  |
|  | TLE4 (CHCHD2P9) | Hancock DB [89] | 9q21.31 | rs2378383 |  |
|  | C11orf30 | Ferreira MA [68] | 11q13.5 | rs7130588 | 2.00E-08 |
|  | T (IL33) (LINC00702) | Tantisira KG [90] | 6q27 | rs6456042 | 6.00E-06 |
|  | GTF3AP1 (IL33) | Torgerson DG [65] | 9p24.1 | rs2381416 | 2.00E-12 |
|  |  | Moffatt MF [11] | 9p24.1 | rs1342326 | 9.00E-10 |
|  |  | Ferreira MA[66] | 9p24.1 | rs72699186 | 2.00E-09 |
|  |  | Ferreira MA [66] | 9p24.1 | rs343496 | 2.00E-06 |
|  |  | Bonnelykke K [72] | 9p24.1 | rs928413 | 9.00E-13 |
|  | BTNL2  (HLA-DRA) | Ramasamy A [71] | 6p21.32 | rs9268516 | 1.00E-08 |
|  | C5orf56 | Wan Yl [69] | 5q31.1 | rs11745587 |  |
|  | EMSY  (C11orf30) |  |  |  |  |
|  | HCG23 | Hirota T | 6p21.32 | rs3117098 | 5.00E-12 |
|  | HLA-DRB9 (HLA-DRA) | Hirota T | 6p21.32 | rs3129890 | 5.00E-13 |
|  | *JRKL-AS1*  *(CNTN5)* | [Leusink M](https://www.ncbi.nlm.nih.gov/pubmed/?term=Leusink%20M%5BAuthor%5D&cauthor=true&cauthor_uid=25963336) [67] | 11q22.1 | rs921561 | 2.56 × 10^−5^ |
|  | MIR8062 | Ding L [81] | 20p12.3 | rs6054973 | 1.00E-07 |
|  | MRPL11P2 | Wan Yl [69] |  |  |  |
|  | (HLA-DQB1) MTCO3P1 | Hirota T [64] | 6p21.32 | rs7775228 | 5.00E-15 |
|  | NNMT | Torgerson DG [65] | 11q23.2 | rs11214966 | 6.00E-07 |
|  | SLC8A1-AS1  (THUMPD2) | Ding L [81] | 2p22.1 | rs6721181 | 6.00E-07 |
|  | SRIP1 | Ding L [81] | 4q12 | rs17218161 | 2.00E-08 |
|  | ACO1 | Wan YI [69] | 9p21.1 | rs10970976 | 4.00E-06 |
|  | BTNL2  (HLA-DRA) | Ramasamy A [71] | 6p21.32 | rs9268516 | 1.00E-08 |
|  | GAPDHP72  (T) | Tantisira KG [90] | 6q27 | rs6456042 | 6.00E-06 |
|  | GTF3AP1 (IL33) | Bonnelykke K [72] | 9p24.1 | rs928413 | 9.00E-13 |
|  | GTF3AP1(RANBP6) | Torgerson DG [65] | 9p24.1 | rs2381416 | 2.00E-12 |
|  | GTF3AP1(RANBP6) | Moffatt MF [11] | 9p24.1 | rs1342326 | 9.00E-10 |
|  | GTF3AP1(RANBP6) | Ferreira MA [66] | 9p24.1 | rs72699186 | 2.00E-09 |
|  | GTF3AP1(RANBP6) | Ferreira MA [66] | 9p24.1 | rs343496 | 2.00E-06 |
|  | HLA-DQB1  (MTCO3P1) | Hirota T [64] | 6p21.32 | rs7775228 | 5.00E-15 |
|  | IL1RL1 (IL18R1) | Wan YI [69] | 2q12.1 | rs9807989 | 6.00E-08 |
|  | RNU1-21P | Kim JH [77] | 11q24.1 | rs17744026 | 3.00E-06 |
|  | THUMPD2  (SLC8A1-AS1) | Ding L [81] | 2p22.1 | rs6721181 | 6.00E-07 |

List of the selected genes including 131 genes, with theSNPs identified and the location on the chromosome and refrences.
