# Supplementary material for: Next Generation Exome Sequencing of Pediatric Asthma Identifies Rare and Novel Variants in Candidate Genes
Source: Dis Markers. 2021 Feb 8;2021:8884229. doi: 10.1155/2021/8884229 (PMC7888305; doi:10.1155/2021/8884229)
Supplement: Supplementary 2 — Table 2-3: gene coverage for 107 genes from the asthma gene panel. [file 8884229.f2.docx]

Additional File 2

Table 2: Gene coverage for 107 genes from the asthma gene panel.

| **Gene** | **1x** | **5x** | **10x** | **20x** | **30x** | **50x** |
| --- | --- | --- | --- | --- | --- | --- |
| ***ABI3BP*** | 0.996 | 0.990 | 0.930 | 0.773 | 0.662 | 0.417 |
| ***ACO1*** | 0.996 | 0.996 | 0.993 | 0.953 | 0.944 | 0.812 |
| ***ADAMTS9*** | 0.997 | 0.957 | 0.953 | 0.899 | 0.821 | 0.647 |
| ***AGFG1*** | 0.997 | 0.884 | 0.830 | 0.772 | 0.670 | 0.319 |
| ***ARPP21*** | 0.996 | 0.995 | 0.989 | 0.881 | 0.840 | 0.685 |
| ***ATG3*** | 0.996 | 0.932 | 0.812 | 0.673 | 0.435 | 0.279 |
| ***BRD2*** | 0.998 | 0.987 | 0.958 | 0.886 | 0.840 | 0.774 |
| ***C5orf51*** | 0.996 | 0.989 | 0.947 | 0.775 | 0.533 | 0.293 |
| ***C6orf118*** | 0.997 | 0.996 | 0.993 | 0.950 | 0.781 | 0.543 |
| ***CDH13*** | 0.996 | 0.996 | 0.990 | 0.927 | 0.825 | 0.659 |
| ***CDHR3*** | 0.996 | 0.989 | 0.971 | 0.951 | 0.889 | 0.751 |
| ***CDK2*** | 0.996 | 0.996 | 0.989 | 0.944 | 0.835 | 0.688 |
| ***CLEC16A*** | 0.996 | 0.959 | 0.938 | 0.881 | 0.799 | 0.685 |
| ***CNTN5*** | 0.996 | 0.994 | 0.934 | 0.849 | 0.720 | 0.603 |
| ***COL22A1*** | 0.996 | 0.980 | 0.955 | 0.914 | 0.841 | 0.682 |
| ***CRB1*** | 0.998 | 0.989 | 0.983 | 0.925 | 0.875 | 0.728 |
| ***CRCT1*** | 0.998 | 0.611 | 0.282 | 0.137 | 0.073 | 0.000 |
| ***CRIM1*** | 0.997 | 0.994 | 0.977 | 0.973 | 0.947 | 0.889 |
| ***DCLK1*** | 0.996 | 0.996 | 0.995 | 0.984 | 0.925 | 0.713 |
| ***ERBB4*** | 0.996 | 0.996 | 0.990 | 0.885 | 0.772 | 0.518 |
| ***FAM19A2*** | 0.995 | 0.995 | 0.993 | 0.939 | 0.758 | 0.747 |
| ***GAB1*** | 0.998 | 0.937 | 0.863 | 0.703 | 0.602 | 0.519 |
| ***GAS1*** | 0.999 | 0.260 | 0.224 | 0.161 | 0.012 | 0.000 |
| ***GAS8*** | 0.997 | 0.964 | 0.910 | 0.838 | 0.771 | 0.539 |
| ***GC*** | 0.996 | 0.996 | 0.994 | 0.972 | 0.897 | 0.807 |
| ***GSDMA*** | 0.996 | 0.995 | 0.989 | 0.938 | 0.835 | 0.686 |
| ***GSDMB*** | 0.997 | 0.996 | 0.996 | 0.995 | 0.987 | 0.919 |
| ***HLA-DOA*** | 0.997 | 0.992 | 0.967 | 0.832 | 0.798 | 0.634 |
| ***HLA-DPA1*** | 0.997 | 0.997 | 0.997 | 0.997 | 0.994 | 0.981 |
| ***HLA-DQA2*** | 0.997 | 0.718 | 0.571 | 0.542 | 0.539 | 0.519 |
| ***HLA-DQB1*** | 0.995 | 0.707 | 0.516 | 0.466 | 0.378 | 0.244 |
| ***HLA-DRA*** | 0.996 | 0.995 | 0.986 | 0.915 | 0.873 | 0.593 |
| ***HLA-DRB1*** | 0.995 | 0.286 | 0.223 | 0.065 | 0.000 | 0.000 |
| ***HPSE2*** | 0.996 | 0.996 | 0.994 | 0.989 | 0.957 | 0.814 |
| ***IGSF3*** | 0.998 | 0.397 | 0.338 | 0.294 | 0.287 | 0.229 |
| ***IKZF1*** | 0.997 | 0.997 | 0.996 | 0.933 | 0.834 | 0.593 |
| ***IKZF3*** | 0.997 | 0.911 | 0.910 | 0.891 | 0.795 | 0.545 |
| ***IKZF4*** | 0.997 | 0.996 | 0.975 | 0.892 | 0.774 | 0.538 |
| ***IL13*** | 0.996 | 0.995 | 0.995 | 0.993 | 0.969 | 0.816 |
| ***IL18R1*** | 0.997 | 0.997 | 0.994 | 0.938 | 0.707 | 0.472 |
| ***IL1RL1*** | 0.997 | 0.983 | 0.947 | 0.916 | 0.833 | 0.672 |
| ***IL2RB*** | 0.997 | 0.959 | 0.953 | 0.914 | 0.824 | 0.669 |
| ***IL33*** | 0.995 | 0.898 | 0.848 | 0.844 | 0.832 | 0.692 |
| ***IL5*** | 0.996 | 0.996 | 0.996 | 0.986 | 0.811 | 0.157 |
| ***IL6R*** | 0.996 | 0.996 | 0.990 | 0.884 | 0.669 | 0.577 |
| ***INSR*** | 0.997 | 0.966 | 0.959 | 0.836 | 0.797 | 0.702 |
| ***IRF1*** | 0.997 | 0.996 | 0.995 | 0.990 | 0.820 | 0.739 |
| ***JRKL*** | 0.999 | 0.999 | 0.998 | 0.995 | 0.974 | 0.725 |
| ***KLHL5*** | 0.997 | 0.994 | 0.945 | 0.846 | 0.720 | 0.428 |
| ***KRT25*** | 0.997 | 0.918 | 0.911 | 0.814 | 0.690 | 0.465 |
| ***LCE3E*** | 0.998 | 0.719 | 0.597 | 0.595 | 0.511 | 0.000 |
| ***LMO4*** | 0.996 | 0.992 | 0.913 | 0.783 | 0.780 | 0.731 |
| ***LRRC32*** | 0.999 | 0.943 | 0.942 | 0.908 | 0.839 | 0.777 |
| ***LRRC3C*** | 0.998 | 0.786 | 0.760 | 0.639 | 0.613 | 0.472 |
| ***MAVS*** | 0.998 | 0.998 | 0.996 | 0.950 | 0.936 | 0.760 |
| ***MKLN1*** | 0.996 | 0.981 | 0.909 | 0.744 | 0.616 | 0.346 |
| ***MTHFR*** | 0.997 | 0.995 | 0.965 | 0.930 | 0.863 | 0.772 |
| ***NOTCH4*** | 0.998 | 0.994 | 0.973 | 0.913 | 0.836 | 0.671 |
| ***OTOGL*** | 0.996 | 0.964 | 0.916 | 0.800 | 0.654 | 0.406 |
| ***OXCT1*** | 0.996 | 0.991 | 0.953 | 0.806 | 0.744 | 0.566 |
| ***PBX2*** | 0.997 | 0.841 | 0.782 | 0.750 | 0.743 | 0.637 |
| ***PDE4D*** | 0.997 | 0.899 | 0.887 | 0.821 | 0.709 | 0.515 |
| ***PRKG1*** | 0.996 | 0.938 | 0.886 | 0.852 | 0.748 | 0.529 |
| ***PSAP*** | 0.996 | 0.996 | 0.989 | 0.939 | 0.923 | 0.776 |
| ***PTCHD3*** | 0.999 | 0.942 | 0.928 | 0.851 | 0.759 | 0.593 |
| ***PTHLH*** | 0.998 | 0.994 | 0.978 | 0.836 | 0.785 | 0.654 |
| ***PYHIN1*** | 0.997 | 0.995 | 0.977 | 0.902 | 0.746 | 0.534 |
| ***RAB18*** | 0.997 | 0.975 | 0.917 | 0.835 | 0.644 | 0.437 |
| ***RAD50*** | 0.997 | 0.973 | 0.943 | 0.841 | 0.613 | 0.310 |
| ***RANBP6*** | 1.000 | 0.701 | 0.665 | 0.622 | 0.581 | 0.483 |
| ***RAP1GAP2*** | 0.996 | 0.992 | 0.965 | 0.879 | 0.761 | 0.543 |
| ***RBM17*** | 0.996 | 0.994 | 0.972 | 0.817 | 0.775 | 0.635 |
| ***RORA*** | 0.996 | 0.940 | 0.754 | 0.744 | 0.734 | 0.542 |
| ***RREB1*** | 0.998 | 0.934 | 0.885 | 0.792 | 0.640 | 0.401 |
| ***SCG3*** | 0.996 | 0.995 | 0.989 | 0.933 | 0.858 | 0.605 |
| ***SCML4*** | 0.997 | 0.997 | 0.996 | 0.994 | 0.976 | 0.795 |
| ***SEMA3E*** | 0.997 | 0.995 | 0.988 | 0.910 | 0.756 | 0.555 |
| ***SGMS1*** | 0.997 | 0.997 | 0.997 | 0.986 | 0.942 | 0.787 |
| ***SLC22A5*** | 0.997 | 0.959 | 0.875 | 0.724 | 0.657 | 0.410 |
| ***SLC25A46*** | 0.997 | 0.955 | 0.856 | 0.730 | 0.589 | 0.273 |
| ***SLC30A8*** | 0.996 | 0.996 | 0.995 | 0.985 | 0.954 | 0.731 |
| ***SLC8A1*** | 0.998 | 0.998 | 0.995 | 0.973 | 0.880 | 0.720 |
| ***SMAD3*** | 0.997 | 0.832 | 0.772 | 0.761 | 0.688 | 0.427 |
| ***SPATS2L*** | 0.997 | 0.997 | 0.996 | 0.982 | 0.881 | 0.633 |
| ***SYNPO2*** | 0.999 | 0.998 | 0.991 | 0.953 | 0.900 | 0.743 |
| ***TBC1D4*** | 0.997 | 0.981 | 0.945 | 0.816 | 0.743 | 0.623 |
| ***THUMPD2*** | 0.996 | 0.991 | 0.811 | 0.674 | 0.550 | 0.422 |
| ***TLN1*** | 0.999 | 0.972 | 0.953 | 0.918 | 0.904 | 0.857 |
| ***TLR1*** | 1.000 | 0.802 | 0.802 | 0.799 | 0.774 | 0.642 |
| ***TNS1*** | 0.997 | 0.989 | 0.978 | 0.902 | 0.847 | 0.719 |
| ***TSLP*** | 0.997 | 0.997 | 0.997 | 0.993 | 0.975 | 0.842 |
| ***TYRP1*** | 0.997 | 0.997 | 0.990 | 0.855 | 0.815 | 0.602 |
| ***VAV3*** | 0.996 | 0.971 | 0.966 | 0.884 | 0.700 | 0.468 |
| ***WDR36*** | 0.997 | 0.977 | 0.942 | 0.832 | 0.688 | 0.470 |
| ***XKR6*** | 0.998 | 0.855 | 0.781 | 0.647 | 0.529 | 0.473 |
| ***XPR1*** | 0.997 | 0.959 | 0.909 | 0.869 | 0.817 | 0.593 |
| ***ZFYVE28*** | 0.997 | 0.903 | 0.873 | 0.729 | 0.573 | 0.325 |
| ***ZNF154*** | 0.998 | 0.959 | 0.949 | 0.904 | 0.882 | 0.749 |
| ***ZNF30*** | 0.998 | 0.998 | 0.998 | 0.982 | 0.804 | 0.520 |
| ***ZNF665*** | 0.999 | 0.998 | 0.980 | 0.906 | 0.794 | 0.566 |
| ***ZNF71*** | 1.000 | 0.924 | 0.833 | 0.699 | 0.471 | 0.409 |
| ***ZNF766*** | 0.998 | 0.996 | 0.980 | 0.879 | 0.779 | 0.753 |

Different coverage depths (1X, 5X,10X, 30X,.), indicate the average times of reads of sequencing occurred per nucleotide in different genes describing the depth of sequencing and the statistical power of the resulted data.
