# Supplementary material for: Next Generation Exome Sequencing of Pediatric Asthma Identifies Rare and Novel Variants in Candidate Genes
Source: Dis Markers. 2021 Feb 8;2021:8884229. doi: 10.1155/2021/8884229 (PMC7888305; doi:10.1155/2021/8884229)
Supplement: Supplementary 3 — Table 3-3: list of selected genes associated with asthma. [file 8884229.f3.docx]

Additional File 3

Table 3 : list of selected genes associated with asthma

| **Number** | **Gene** | **FIRST AUTHOR** | **REGION** | **SNPS** | **p-value** |
| --- | --- | --- | --- | --- | --- |
|  | GAB1 | Hirota T [64] | 4q31.21 | rs3805236 | 7.00E-08 |
|  | TSLP | Hirota T [64] | 5q22.1 | rs1837253 | 1.00E-16 |
|  |  | Torgerson DG [65] | 5q22.1 | rs1837253 | 1.00E-14 |
|  |  | Ferreira MA [66] | 5q22.1 | rs1837253 | 1.00E-09 |
|  | PBX2 | Hirota T [64] | 6p21.32 | rs204993 | 2.00E-15 |
|  | NOTCH4 | Hirota T [64] | 6p21.32 | rs404860 | 4.00E-23 |
|  | BRD2 | Hirota T [64] | 6p21.32 | rs9500927 | 4.00E-09 |
|  |  | [Leusink M](https://www.ncbi.nlm.nih.gov/pubmed/?term=Leusink%20M%5BAuthor%5D&cauthor=true&cauthor_uid=25963336) [67] | 6p21.32 | rs10484568 |  |
|  |  | [Leusink M](https://www.ncbi.nlm.nih.gov/pubmed/?term=Leusink%20M%5BAuthor%5D&cauthor=true&cauthor_uid=25963336) [67] | 6p21.32 | rs10484568 |  |
|  | CDK2 | Hirota T [64] | 12q13.2 | rs2069408 | 1.00E-10 |
|  | IKZF4 | Hirota T [64] | 12q13.2 | rs1701704 | 2.00E-13 |
|  | SLC30A8 | Noguchi E [68] | 8q24.11 | rs3019885 | 5.00E-13 |
|  | IL18R1 | Moffatt MF [11] | 2q12.1 | rs3771166 | 3.00E-09 |
|  |  | Wan YI [69] | 2q12.1 | rs9807989 | 6.00E-08 |
|  |  | Barreto-Luis A [70] | 2q12.1 | rs10197862 | 2.00E-06 |
|  |  | Ramasamy A [71] | 2q12.1 | rs13408661 | 1.00E-09 |
|  | PRKG1 | Ferreira MA [68] | 10q21.1 | rs7922491 | 5.00E-07 |
|  | IL6R | Ferreira MA [68] | 1q21.3 | rs4129267 | 2.00E-08 |
|  | SMAD3 | Moffatt MF [11] | 15q22.33 | rs744910 | 4.00E-09 |
|  |  | Ferreira MA [66] | 15q22.33 | rs17294280 | 4.00E-09 |
|  | GSDMA | Moffatt MF [11] | 17q21.1 | rs3894194 | 5.00E-09 |
|  |  | Ferreira MA [66] | 17q21.1 | rs7212938 | 4.00E-10 |
|  |  | Bonnelykke K [72] | 17q21.1 | rs3894194 | 3.00E-21 |
|  | IL2RB | Moffatt MF [11] | 22q12.3 | rs2284033 | 1.00E-08 |
|  | SLC22A5 | Moffatt MF [11] | 5q31.1 | rs2073643 | 2.00E-07 |
|  | IL13 | Moffatt MF [11] | 5q31.1 | rs1295686 | 1.00E-07 |
|  |  | Bonnelykke K [72] | 5q31.1 | rs1295686 | 2.00E-06 |
|  | RORA | Moffatt MF [11] | 15q22.2 | rs11071559 | 1.00E-07 |
|  | LRRC3C | Ferreira MA [73] | 17q21.1 | rs6503525 | 5.00E-07 |
|  |  | Wan YI [69] | 17q21.1 | rs4794820 | 1.00E-08 |
|  | ACO1 | Wan YI [69] | 9p21.1 | rs10970976 | 4.00E-06 |
|  | ZNF665 | Wan YI [69] | 19q13.42 | rs16984547 | 4.00E-06 |
|  | CRB1 | Sleiman PM [74] | 1q31.3 | rs2786098 | 2.00E-13 |
|  |  | Sleiman PM [74] | 1q31.3 | rs2786098 | 9.00E-11 |
|  | RAD50 | Li X [75] | 5q31.1 | rs2244012 | 3.00E-07 |
|  |  | Bonnelykke K [72] | 5q31.1 | rs6871536 | 8.00E-07 |
|  | SCG3 | Li X [75] | 15q21.2 | rs17525472 | 2.00E-06 |
|  | SPATS2L | Himes BE [76] | 2q33.1 | rs295137 | 1.00E-06 |
|  | ADAMTS9 | Barreto-Luis A [70] | 3p14.1 | rs9866261 | 1.00E-07 |
|  | CRIM1 | Kim JH [77] | 2p22.2 | rs848512 | 1.00E-06 |
|  | ZNF71 | Kim JH [77] | 19q13.43 | rs10404342 | 8.00E-06 |
|  | TLN1 | Kim JH [77] | 9p13.3 | rs4879926 | 8.00E-06 |
|  | SYNPO2 | Kim JH [77] | 4q26 | rs1472066 | 8.00E-06 |
|  | LCE3E | Torgerson DG [65] | 1q21.3 | rs4845783 | 6.00E-06 |
|  | CDH13 | Kim JH [77] | 16q23.3 | rs6563898 | 8.00E-06 |
|  | PDE4D | Himes BE [78] | 5q12.1 | rs1588265 | 3.00E-08 |
|  | PYHIN1 | Torgerson DG [65] | 1q23.1 | rs1101999 | 4.00E-09 |
|  | RANBP6 | Torgerson DG [65] | 9p24.1 | rs2381416 | 2.00E-12 |
|  |  | Moffatt MF [11] | 9p24.1 | rs1342326 | 9.00E-10 |
|  |  | Ferreira MA [66] | 9p24.1 | rs72699186 | 2.00E-09 |
|  |  | Ferreira MA [66] | 9p24.1 | rs343496 | 2.00E-06 |
|  | CRCT1 | Torgerson DG [65] | 1q21.3 | rs4845783 | 6.00E-06 |
|  | HLA-DPA1 | Noguchi E [79] | 6p21.32 | rs987870 | 2.00E-10 |
|  | IRF1 | Myers RA [80] | 5q31.1 | rs2549003 | 9.00E-07 |
|  | MAVS | Li X [75] | 20p13 | rs4815617 | 8.00E-06 |
|  | ERBB4 | Myers RA [80] | 2q34 | rs4673659 | 9.00E-07 |
|  | C6orf118 | Myers RA [80] | 6q27 | rs2675724 | 2.00E-07 |
|  | RAP1GAP2 | Myers RA [80] | 17p13.3 | rs9895098 | 3.00E-07 |
|  | TLR1 | Ferreira MA [66] | 4p14 | rs4833095 | 5.00E-12 |
|  | WDR36 | Ferreira MA [66] | 5q22.1 | rs1438673 | 3.00E-11 |
|  | CLEC16A | Ferreira MA [66] | 16p13.13 | rs62026376 | 1.00E-08 |
|  | SLC25A46 | Ferreira MA [66] | 5q22.1 | rs3853750 | 2.00E-07 |
|  | PTHLH | Ferreira MA [66] | 12p11.22 | rs11049300 | 3.00E-07 |
|  | IKZF3 | Ferreira MA[66] | 17q21.1 | rs12450323 | 4.00E-07 |
|  | XKR6 | Ferreira MA [66] | 8p23.1 | rs6982751 | 4.00E-07 |
|  | RBM17 | Ferreira MA [66] | 10p15.1 | rs41295115 | 5.00E-07 |
|  | TNS1 | Ferreira MA [66] | 2q35 | rs76043829 | 6.00E-07 |
|  | VAV3 | Ferreira MA [66] | 1p13.3 | rs7521681 | 7.00E-07 |
|  | GAS1 | Ding L [81] | 9q21.33 | rs11141597 | 2.00E-06 |
|  | FAM19A2 | Ferreira MA[66] | 12q14.1 | rs17605016 | 2.00E-06 |
|  | HLA-DRB1 | Lasky-Su J [82] | 6p21.32 | rs9272346 | 2.00E-08 |
|  | COL22A1 | Duan QL [83] | 8q24.23 | rs6988229 | 9.00E-06 |
|  | RAB18 | White MJ [84] | 10p12.1 | rs660498 | 2.00E-07 |
|  | DCLK1 | Forno E [85] | 13q13.3 | rs7328278 | 3.00E-06 |
|  | PTCHD3 | White MJ [84] | 10p12.1 | rs660498 | 2.00E-07 |
|  | SEMA3E | White MJ [84] | 7q21.11 | rs17446324 | 5.00E-06 |
|  | INSR | White MJ [84] | 19p13.2 | rs67731056 | 7.00E-06 |
|  | TYRP1 | Ding L [81] | 9p23 | rs16929097 | 8.00E-09 |
|  | CDHR3 | Bonnelykke K [72] | 7q22.3 | rs6967330 | 3.00E-14 |
|  | IGSF3 | Ding L [81] | 1p13.1 | rs17036023 | 5.00E-06 |
|  | HPSE2 | Ding L [81] | 10q24.2 | rs12570188 | 5.00E-08 |
|  | PSAP | Ding L [81] | 10q22.1 | rs11000019 | 8.00E-08 |
|  | ATG3 | Ding L [81] | 3q13.2 | rs2705520 | 2.00E-06 |
|  | ARPP21 | Ding L [81] | 3p22.3 | rs17033506 | 4.00E-07 |
|  | SLC8A1 | Ding L [81] | 2p22.1 | rs6721181 | 6.00E-07 |
|  | THUMPD2 | Ding L [81] | 2p22.1 | rs6721181 | 6.00E-07 |
|  | MKLN1 | Ding L [81] | 7q32.3 | rs7807274 | 4.00E-06 |
|  | XPR1 | Ding L [81] | 1q25.3 | rs7527074 | 9.00E-06 |
|  | IL33 | Torgerson DG [65] | 9p24.1 | rs2381416 | 2.00E-12 |
|  |  | Moffatt MF [11] | 9p24.1 | rs1342326 | 9.00E-10 |
|  |  | Ferreira MA [66] | 9p24.1 | rs72699186 | 2.00E-09 |
|  |  | Bonnelykke K [72] | 9p24.1 | rs928413 | 9.00E-13 |
|  | ABI3BP | Ding L [81] | 3q12.2 | rs9823506 | 6.00E-08 |
|  | KLHL5 | Ding L [81] | 4p14 | rs35141484 | 3.00E-07 |
|  | JRKL | [Leusink M](https://www.ncbi.nlm.nih.gov/pubmed/?term=Leusink%20M%5BAuthor%5D&cauthor=true&cauthor_uid=25963336) [67] |  | rs921561 | 2.56 × 10^−5^ |
|  | OXCT1 | [Leusink M](https://www.ncbi.nlm.nih.gov/pubmed/?term=Leusink%20M%5BAuthor%5D&cauthor=true&cauthor_uid=25963336) [67] |  | rs151191974 |  |
|  | AGFG1 | Himes BE | 2q36.3 | rs6731443 | 2.00E-06 |
|  | GC | Lasky-Su J [86] | 4q13.3 | rs2282679 | 2.00E-14 |
|  | *RREB1* | [Leusink M](https://www.ncbi.nlm.nih.gov/pubmed/?term=Leusink%20M%5BAuthor%5D&cauthor=true&cauthor_uid=25963336) [67] |  | rs35742417 | 2.44 × 10^−6^ |
|  | *GAS8* |  |  | rs117053233 | 2.63 × 10−5 6.14 × 10−5 |
|  | *KRT25* | [Leusink M](https://www.ncbi.nlm.nih.gov/pubmed/?term=Leusink%20M%5BAuthor%5D&cauthor=true&cauthor_uid=25963336) [67] |  | rs72821893 | 6.21 × 10−5 |
|  | *IKZF1* | [Leusink M](https://www.ncbi.nlm.nih.gov/pubmed/?term=Leusink%20M%5BAuthor%5D&cauthor=true&cauthor_uid=25963336) [67] |  | rs1456896 | 8.80 × 10−5 |
|  | *CNTN5* | [Leusink M](https://www.ncbi.nlm.nih.gov/pubmed/?term=Leusink%20M%5BAuthor%5D&cauthor=true&cauthor_uid=25963336) [67] |  | rs921561 | 2.56 × 10^−5^ |
|  | *OTOGL* | [Leusink M](https://www.ncbi.nlm.nih.gov/pubmed/?term=Leusink%20M%5BAuthor%5D&cauthor=true&cauthor_uid=25963336) [67] |  | rs1551120 | 2.67 × 10−5 |
|  | *TBC1D4* | [Leusink M](https://www.ncbi.nlm.nih.gov/pubmed/?term=Leusink%20M%5BAuthor%5D&cauthor=true&cauthor_uid=25963336) [67] |  | rs716655 | 5.56 × 10−5 |
|  | *SCML4* | [Leusink M](https://www.ncbi.nlm.nih.gov/pubmed/?term=Leusink%20M%5BAuthor%5D&cauthor=true&cauthor_uid=25963336) [67] |  | rs847005 | 4.49 × 10−5 |
|  | *ZNF30* | [Leusink M](https://www.ncbi.nlm.nih.gov/pubmed/?term=Leusink%20M%5BAuthor%5D&cauthor=true&cauthor_uid=25963336) [67] |  | rs142299823 | 4.60 × 10−5 |
|  | *SGMS1* | [Leusink M](https://www.ncbi.nlm.nih.gov/pubmed/?term=Leusink%20M%5BAuthor%5D&cauthor=true&cauthor_uid=25963336) [67] |  | rs2574951 | 4.68 × 10−5 |
|  | *LMO4* | [Leusink M](https://www.ncbi.nlm.nih.gov/pubmed/?term=Leusink%20M%5BAuthor%5D&cauthor=true&cauthor_uid=25963336) [67] |  | rs4655852 |  |
|  | *ZNF766* | [Leusink M](https://www.ncbi.nlm.nih.gov/pubmed/?term=Leusink%20M%5BAuthor%5D&cauthor=true&cauthor_uid=25963336) [67] |  | rs12462608 | 5.18 × 10−5 |
|  | *C5orf51* | [Leusink M](https://www.ncbi.nlm.nih.gov/pubmed/?term=Leusink%20M%5BAuthor%5D&cauthor=true&cauthor_uid=25963336) [67] |  | rs151191974 | 6.44 × 10−5 |
|  | *ZNF154* | [Leusink M](https://www.ncbi.nlm.nih.gov/pubmed/?term=Leusink%20M%5BAuthor%5D&cauthor=true&cauthor_uid=25963336) [67] |  | rs34282745 | 8.13 × 10−5 |
|  | *ZFYVE28* | [Leusink M](https://www.ncbi.nlm.nih.gov/pubmed/?term=Leusink%20M%5BAuthor%5D&cauthor=true&cauthor_uid=25963336) [67] |  | rs17768776 | 8.69 × 10−5 |
|  | MTHFR | [Igartua C](https://www.ncbi.nlm.nih.gov/pubmed/?term=Igartua%20C%5BAuthor%5D&cauthor=true&cauthor_uid=25591454) [87] |  | rs1801133 | 9.30 × 10−5 |
|  | IL5 | Myers RA [80] | 5q31.1 | rs2549003 | 9.00E-07 |
|  | LRRC32 | Ferreira MA [68] | 11q13.5 | rs7130588 | 2.00E-08 |
|  | GSDMB | Moffatt MF [11] | 17q21.1 | rs7216389 | 9.00E-11 |
|  |  | Moffatt MF [11] | 17q21.1 | rs2305480 | 1.00E-07 |
|  |  | Torgerson DG [65] | 17q21.1 | rs11078927 | 2.00E-16 |
|  |  | Bonnelykke K [72] | 17q21.1 | rs2305480 | 6.00E-23 |
|  |  |  | 17q12–21 | rs2305480 |  |
|  |  |  | 17q12–21 | rs7216389 |  |
|  | HLA-DOA (BRD2 ) | Hirota T [64] | 6p21.32 | rs9500927 | 4.00E-09 |
|  | HLA-DOA  (BRD2) | [Leusink M](https://www.ncbi.nlm.nih.gov/pubmed/?term=Leusink%20M%5BAuthor%5D&cauthor=true&cauthor_uid=25963336) [67] |  | rs10484568 | 7.42 × 10−5 |
|  | HLA-DQA2 | Hirota T [64] | 6p21.32 | rs9275698 | 5.00E-12 |
|  | HLA-DQA1 (HLA-DQB1) | Moffatt MF [22, 88]. | 6p21.32 | rs9273349 | 7.00E-14 |
|  |  | Ferreira MA [66] | 6p21.32 | rs9273373 | 4.00E-14 |
|  | HLA-DQB1 (MTCO3P1) | Hirota T [64] | 6p21.32 | rs7775228 | 5.00E-15 |
|  | BTNL2  (HLA-DRA) | Ramasamy A [71] | 6p21.32 | rs9268516 | 1.00E-08 |
|  | HLA-DRA (HLA-DRB9) | Hirota T [64] | 6p21.32 | rs3129890 | 5.00E-13 |
|  | IL1RL1 | Ramasamy A [71] | 2q12.1 | rs13408661 | 1.00E-09 |
|  |  | Barreto-Luis A [70] | 2q12.1 | rs10197862 | 2.00E-06 |
|  |  | Torgerson DG [65] | 2q12.1 | rs3771180 | 2.00E-15 |
|  |  | Ferreira MA [66] | 2q12.1 | rs10197862 | 4.00E-11 |
|  | IL1RL1 - IL18R1 | Wan YI [69] | 2q12.1 | rs9807989 | 6.00E-08 |
|  | C20orf29  (AP5S1)(MAVS ) | Li X [75] | 20p13 | rs4815617 | 8.00E-06 |
|  | BCL2  (BCLAF1P1  ) |  |  |  |  |
|  | TLE4 (CHCHD2P9) | Hancock DB [89] | 9q21.31 | rs2378383 |  |
|  | C11orf30 | Ferreira MA [68] | 11q13.5 | rs7130588 | 2.00E-08 |
|  | T (IL33) (LINC00702) | Tantisira KG [90] | 6q27 | rs6456042 | 6.00E-06 |
|  | GTF3AP1 (IL33) | Torgerson DG [65] | 9p24.1 | rs2381416 | 2.00E-12 |
|  |  | Moffatt MF [11] | 9p24.1 | rs1342326 | 9.00E-10 |
|  |  | Ferreira MA[66] | 9p24.1 | rs72699186 | 2.00E-09 |
|  |  | Ferreira MA [66] | 9p24.1 | rs343496 | 2.00E-06 |
|  |  | Bonnelykke K [72] | 9p24.1 | rs928413 | 9.00E-13 |
|  | BTNL2  (HLA-DRA) | Ramasamy A [71] | 6p21.32 | rs9268516 | 1.00E-08 |
|  | C5orf56 | Wan Yl [69] | 5q31.1 | rs11745587 |  |
|  | EMSY  (C11orf30) |  |  |  |  |
|  | HCG23 | Hirota T | 6p21.32 | rs3117098 | 5.00E-12 |
|  | HLA-DRB9 (HLA-DRA) | Hirota T | 6p21.32 | rs3129890 | 5.00E-13 |
|  | *JRKL-AS1*  *(CNTN5)* | [Leusink M](https://www.ncbi.nlm.nih.gov/pubmed/?term=Leusink%20M%5BAuthor%5D&cauthor=true&cauthor_uid=25963336) [67] | 11q22.1 | rs921561 | 2.56 × 10^−5^ |
|  | MIR8062 | Ding L [81] | 20p12.3 | rs6054973 | 1.00E-07 |
|  | MRPL11P2 | Wan Yl [69] |  |  |  |
|  | (HLA-DQB1) MTCO3P1 | Hirota T [64] | 6p21.32 | rs7775228 | 5.00E-15 |
|  | NNMT | Torgerson DG [65] | 11q23.2 | rs11214966 | 6.00E-07 |
|  | SLC8A1-AS1  (THUMPD2) | Ding L [81] | 2p22.1 | rs6721181 | 6.00E-07 |
|  | SRIP1 | Ding L [81] | 4q12 | rs17218161 | 2.00E-08 |
|  | ACO1 | Wan YI [69] | 9p21.1 | rs10970976 | 4.00E-06 |
|  | BTNL2  (HLA-DRA) | Ramasamy A [71] | 6p21.32 | rs9268516 | 1.00E-08 |
|  | GAPDHP72  (T) | Tantisira KG [90] | 6q27 | rs6456042 | 6.00E-06 |
|  | GTF3AP1 (IL33) | Bonnelykke K [72] | 9p24.1 | rs928413 | 9.00E-13 |
|  | GTF3AP1(RANBP6) | Torgerson DG [65] | 9p24.1 | rs2381416 | 2.00E-12 |
|  | GTF3AP1(RANBP6) | Moffatt MF [11] | 9p24.1 | rs1342326 | 9.00E-10 |
|  | GTF3AP1(RANBP6) | Ferreira MA [66] | 9p24.1 | rs72699186 | 2.00E-09 |
|  | GTF3AP1(RANBP6) | Ferreira MA [66] | 9p24.1 | rs343496 | 2.00E-06 |
|  | HLA-DQB1  (MTCO3P1) | Hirota T [64] | 6p21.32 | rs7775228 | 5.00E-15 |
|  | IL1RL1 (IL18R1) | Wan YI [69] | 2q12.1 | rs9807989 | 6.00E-08 |
|  | RNU1-21P | Kim JH [77] | 11q24.1 | rs17744026 | 3.00E-06 |
|  | THUMPD2  (SLC8A1-AS1) | Ding L [81] | 2p22.1 | rs6721181 | 6.00E-07 |

List of the selected genes in the study, including 131 genes, with the identified SNPs reported and the location on the chromosome and references.
